# Numerical Design of Granular Support for Three-Way Catalyzed Solid- and Porous-Particles Membrane Filters

**DOI:** 10.3390/membranes13070644

**Published:** 2023-07-04

**Authors:** Teerapat Suteerapongpun, Katsunori Hanamura

**Affiliations:** Department of Mechanical Engineering, Tokyo Institute of Technology, 2-12-1 Ookayama, Meguro City, Tokyo 152-8550, Japan; hanamura.k.aa@m.titech.ac.jp

**Keywords:** macroporous, three-way catalyze (twc), particulate filter, membrane filter, pressure drop

## Abstract

A granular substrate used as a support for a three-way catalyzed (TWC) solid-particle membrane filter was investigated through numerical simulation. The proposed support could reduce the amount of required catalyst material by 39% and lower the pressure drop by 33%, compared to a conventional filter, while achieving almost 100% soot-filtration. Moreover, TWC porous particles, which are designed to introduce a fluid flow into their interconnected pore network, further decrease the pressure drop. However, a trade-off exists between the amount of the introduced fluid flow and the specific surface area.

## 1. Introduction

The new Euro 7 Emission Standards [1] will be adopted by many countries in the world, including Japan. They will require stricter regulations on car emissions, particularly focusing on reducing particulate matter (PM) levels, including particle mass (PMs) and particle numbers (PNs) [2]. In the current gasoline after-treatment systems, the primary device used to capture PM is a gasoline particulate filter (GPF). Currently, the GPF faces two main challenges, poor filtration efficiency when the filter is empty and increased pressure drop caused by accumulated PM inside the filter. Poor filtration efficiency arises only when the filter is completely clean, and it disappears as PM starts accumulating on the filter. However, the problem of increasing pressure drop due to PM clogging requires the removal of deposited PM. This process, known as filter regeneration, can result in higher fuel consumption.

**Figure 1:**
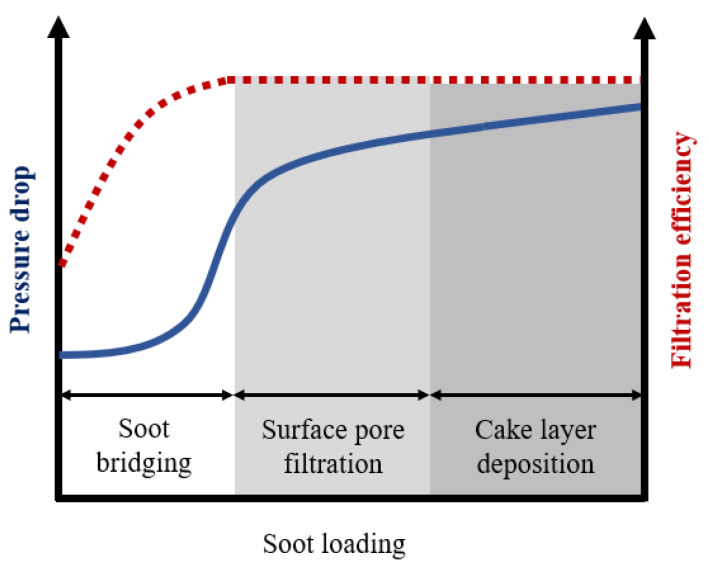
Increase in pressure drop of soot trapping on a conventional particulate filter. It shows a 3-phase characteristic, including soot-bridge formation, surface pore filtration, and soot cake layer formation. After completing the bridging phase, the inflection point in the pressure drop graph, the filtration efficiency becomes almost 100%, as shown by the dashed line.

**Figure 2:**
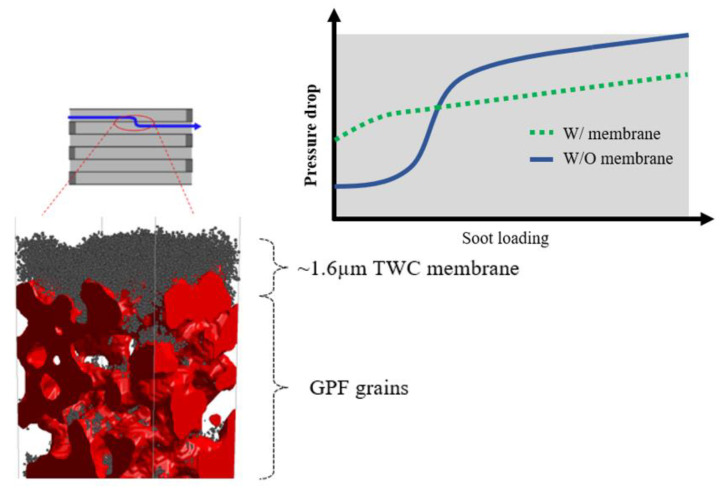
A schematic of a TWC membrane layer coated on a conventional GPF wall (**left**), and the pressure drop during soot trapping with and without a membrane (**right**).

A previous study revealed a pressure drop increase due to PM deposition inside a granular filter [3]. It showed the characteristic s-curve of pressure drop increase, illustrating three phases, soot-bridge formation, surface pore filtration, and soot cake layer formation, as shown in Figure 1. The most drastic pressure drop increase occurred during the bridge formation and pore filtration phases [4], where soot accumulates inside the pores until completely filling them. Consequently, to address the issue of the drastic increase in pressure drop, a novel approach was proposed involving the use of a three-way catalyzed (TWC) membrane [5]. This membrane, comprised of sub-micron-scale particles, was fabricated onto a conventional GPF. Introduction of a membrane has been shown to prevent soot deposition inside the GPF pores at the initial state because the GPF pores were already filled by the membrane. Thus, utilizing a higher membrane permeability compared to soot yielded a lower pressure in long-term soot trapping compared to a conventional particulate filter [6,7], as shown in Figure 2. Moreover, the membrane could provide almost complete filtration efficiency from the beginning owing to pre-loaded membrane particles in the particulate filter pores, overcoming this limitation of the conventional particulate filter [4].

Despite the high-filtration efficiency of the membrane, there is one inevitable trade-off. There is a higher initial pressure drop from the membrane layer because of the pre-loaded sub-micrometer scale particles. A previous study showed a drastic increase in pressure drop during membrane fabrication on a conventional particulate filter. This occurred in the bridging and pore deposition phases, similar to soot trapping behavior [5,7,8]. As a result, it reflects that a conventional GPF is not the best-optimized support for a membrane filter. Hence, this study utilized a computer simulation to develop a granular substrate designed explicitly to support a TWC membrane. This study investigated two main parameters, support thickness and pore size. Thus, simulations of membrane fabrication on several thin granular supports with a small pore size have been conducted, targeting the lowest pressure drop after fabricating the membrane.

Recent research has uncovered an interconnected pore network within microporous TWC particles [9]. Thus, utilizing gas flow through a porous-particle membrane may offer higher permeability and the potential for reducing pressure drop in our TWC membrane. Moreover, an earlier study also demonstrated that adding a porous structure could improve catalytic performance [10], as it provides a larger effective surface area on the outer shell, and internal surface pores enhance the catalytic effect of the TWC. As a result, the TWC membrane was further developed by adding a porous structure inside each particle using a template-assisted spray method with a subsequent additional heating process. Poly(methyl methacrylate) (PMMA) was used as a template [9]. Porous-TWC particles were fabricated on a particulate filter using the methodology of previous studies [6,11]. The porous structure can be classified according to its pore size. Pores smaller than 2 nm are called micropores, those between 2 nm and 50 nm are mesopores, while those larger than 50 nm are macropores. In this study, the porous-particle membrane has a macroporous structure.

This study investigated the properties of macropores within particles that comprise the membrane, in addition to the proportions of PMMA and TWC employed in membrane fabrication. The goal of the current study is to obtain the lowest pressure drop across a porous-particle membrane and introduce more flow fractions inside each particle’s porous structure.

## 2. Simulation Methods

### 2.1. 3D GPF Structure with Modification of the Granular Support for Supporting Membrane

**Figure 3:**
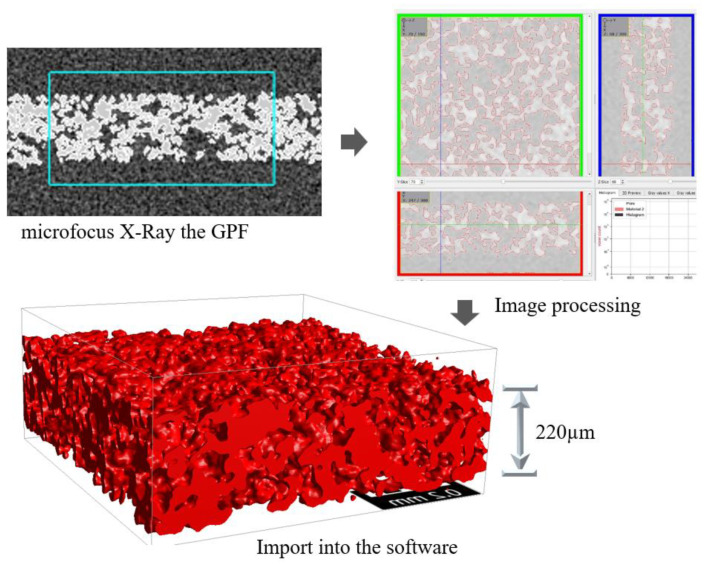
GPF model reconstruction process. GPF image obtained from microfocus X-ray (**top left**), GPF model after reducing noise by image processing (**top right**), and the final 3D GPF model (**bottom**).

Computed tomography was used to scan a commercial gasoline particulate filter (GPF) manufactured by Nippon Gaishi. It was examined using the microfocus X-ray system (inspeXio SMX-225CTS, SHIMADZU). A stack of scanned images was imported and used to construct a 3D model in a modeling software (GeoDict 2022, MATH 2 MARKET). The original resolution was 2.44336 µm per voxel in a domain size of 300 × 300 × 150 voxels with a periodic domain. Noise-cancelling algorithms (non-local means filter) were used to eliminate unwanted noise surrounding the pores and grains of a GPF. The GPF model reconstruction procedure is depicted in Figure 3. The original porosity of the reconstructed GPF in the software (GeoDict 2022, MATH 2 MARKET) was 64%, and the mean-pore size was 22 µm with a thickness of 220 µm. 

The morphology of the artificial granular supporting material was modified from a conventional GPF to maintain a realistic shape and optimize the substrate to support the membrane. This involved resizing each artificial support to achieve the desired pore size and adjusting the depth to achieve the desired thickness. A conventional GPF provides deep pore filtration when fabricating membrane filters, resulting in high material consumption and increased pressure drop. To address these issues, the artificial substrates used in this study were designed with smaller pore sizes to prevent deep particle deposition, thereby minimizing material consumption and pressure drop. As a result, the artificial supports used in this study had pore sizes ranging from 6 µm to 14 µm and a voxel size of 1.33 µm. 

**Figure 4:**
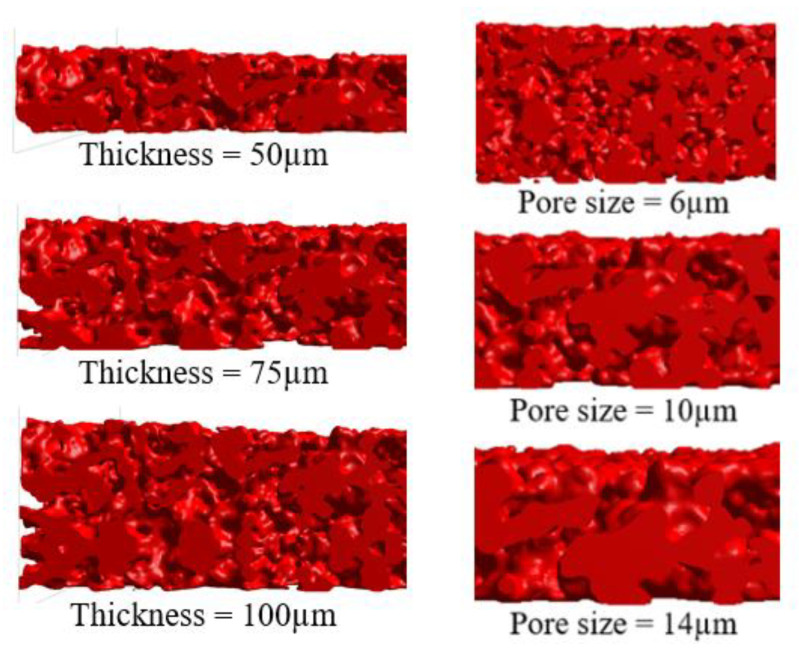
Cross-sectional images of artificial granular substrates with varying thickness (**left column**) and pore size (**right column**).

The support thickness is not necessarily as great as the original GPF, since the membrane demonstrated excellent filtration efficiency from the beginning [12]. Therefore, five thicknesses, 50 µm, 75 µm, 100 µm, 125 µm, and 150 µm were examined. After each membrane fabrication simulation was complete, pressure drop and particle consumption were evaluated. An example of artificial granular substrates for support membranes is shown in Figure 4.

### 2.2. Filtration Simulation Methodology

**Figure 5:**
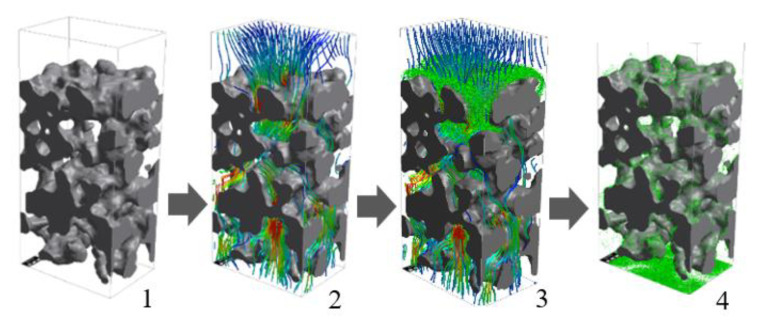
A schematic of a filtering simulation consisting of 4 main processes, 1. definition of a porous structure model, 2. fluid flow computation, 3. particle tracking, and 4. particle clogging.

Simulations of membrane fabrication and soot trapping conducted in this study followed a previous study [5]. Generally, each simulation involves several filtration iterations. One iteration is denoted as a single filtration batch, described in four main processes, 1. definition of a porous structure model, 2. fluid flow computation, 3. particle tracking, and 4. particle clogging, as shown in Figure 5. The following description provides a comprehensive overview of each step:

1. Definition of a porous structure model: The simulation employs a porous structure obtained through either scanned images of a real structure (as outlined in Section 2.1) or generated within the software (GeoDict 2022, MATH 2 MARKET). Subsequently, the substrate’s properties, such as the Hamaker constant, are defined to facilitate subsequent calculations in the particle clogging step.
2. Fluid flow computation: This study adopts a one-way coupling approach for fluid flow computation. The velocity profile was employed for a single batch of particle trapping and recalculated in the subsequent batch, taking into account the presence of trapped particles. Consequently, it is reasonable to assume that the flow is stationary, i.e., it does not change over time. Moreover, the fluid is assumed to be a Newtonian fluid and incompressible due to the moderate flow rate. The fluid flow through the pores is described by the stationary Navier–Stokes equations and the continuity equation shown in the following equations:
  $$\begin{array}{cc} & -\mu ∆\vec{u}+\rho_{f}\left(\vec{u}∙\nabla \right)\vec{u}+\nabla P=\vec{f}\;.\\ \mathrm{And} & \nabla ∙\vec{u}=0\; \end{array}$$
  Here, $\rho_{f}$ represents the fluid density, μ denotes dynamic viscosity, $\vec{u}$ is the velocity vector, P indicates the pressure, and $\vec{f}$ is the external force acting on the fluid.
3. Particle tracking: In this step, particles are tracked through the fluid flow field elucidated in Step 2. Within each batch, particles are treated as independent objects and do not interact with each other. Consequently, a large number of particles could result in significant volume loss due to particle overlap. The movement of particles is governed by the momentum equation, expressed as:
  Particle momentum = stoke drag + external forces.
  Mathematically, this can be written as:
  $$m\frac{d\vec{v}}{dt}=6\pi \mu \frac{R}{C_{c}}\left(\vec{u}-\vec{v}+\sqrt{2D}\frac{Rd\vec{W}\left(t\right)}{C_{c}}\right)+Q\vec{E}+\vec{F}$$
  Here, m represents particle mass, $\vec{v}$ denotes particle velocity, t signifies time, μ is dynamic viscosity, R represents particle radius, $C_{c}$ indicates Cunningham correction [13], $\vec{u}$ represents the fluid velocity, D represents diffusivity, $d\vec{W}$ represents the 3D-Wienber measure, Q represents the particle charge, $\vec{E}$ represents the electric field, and $\vec{F}$ represents the external force, such as the gravity force.
4. Particle clogging: The particle clogging model employed in this study incorporates the Hamaker attraction, a form of Van der Waals attraction. Mathematically, it can be expressed as:
  $$Attraction\;energy=\frac{H_{11}R}{12a_{0}}$$
  Here, *H*_11_ is the Hamaker constant of each pair of particles, *R* is a particle radius, and *a*_0_ is an adhesion distance or equilibrium spacing between the particle and the surface (assumed to have a typical value of 4 Å).

**Figure 6:**
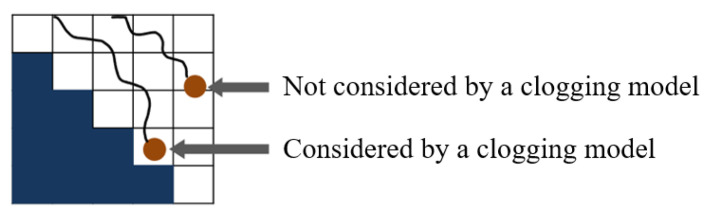
Schematic of the clogging model. Particles entering voxels adjacent to the substrate are trapped by the model, while non-adjacent particles are not.

The clogging model for particles traveling close to the substrate is shown in Figure 6. A particle is captured when its kinetic energy is less than the Hamaker attraction. Thus, the simplified term of the relationship between a particle’s velocity and the Hamaker constant can be expressed as:

$$v^{2}<\frac{H_{11}}{4\pi \rho a_{0}R^{2}}$$

Here *v* is a particle velocity and *ρ* is a particle density.

After each batch, the fluid flow field is recalculated by considering the original porous substrate and new trapped particles, as elucidated in Step 2. Then, the next batch of particles is introduced along with a new flow field, and particle clogging is computationally determined. Pressure drop, filtration efficiency, and the total number of trapped particles are recorded in each batch and summarized at the end of the simulation. The iteration continues until it reaches the targeted total particle trapping.

### 2.3. Simulation Procedure for Membrane Fabrication on Granular Substrates and Soot Trapping on Membranes

Optimization of membrane fabrication on the artificial granular supports was controlled using the same simulation parameters and varying only the substrate model, as shown in Figure 4. The parameters were as follows. TWC particles were used with diameters between 0.9–2.25 µm and a mean diameter of 1.6 µm. The density of TWC particles was 2600 kg/m^3^, measured from a single TWC particle. Most of the electrostatic effects were ignored since the electric field of the GPF substrate was negligible [14,15]. As shown in a previous study, the TWC particle movement was governed by the Stokes equations with Brownian effect influence. The Hamaker constants for TWC-TWC and TWC-GPF particles are 1.52 × 10^−19^ J and 1.92 × 10^−19^ J, respectively [16]. The working fluid was air at 25 °C, density = 1.204 kg/m^3^, dynamic viscosity = 1.834 × 10^−5^ kg/m s, kinematic viscosity = 1.52326 × 10^−5^ m/s^2^. The superficial velocity of the working fluid = 0.51 cm/s.

In the case of soot trapping on the membrane, the diameter of the spherically-shaped soot particles ranged from 30 to 300 nm. According to a previous study, the density of a soot particle depends on its size. Larger diameter particles have lower density [17]. The membrane structure was generated using the membrane fabrication simulation. Then, membrane layers with thicknesses ranging from 15 µm to 55 µm were obtained and adjusted Hamaker constants were 4.7 × 10^−19^ J for soot-soot and 2.67 × 10^−19^ J for soot-TWC. The superficial velocity of the working fluid = 3.4 cm/s. 

### 2.4. Simulation Procedure for Porous-Particle TWC Membrane Formation

In a practical experiment, a porous-particle three-way catalyst (TWC) membrane was fabricated by depositing porous particles ranging in size from 1 µm to 1.5 µm using a membrane fabrication process similar to that described in a previous study [5]. Each of these particles was composed of solid and porous components. The solid component consisted of TWC material in precursor solutions, and the pore component was initially occupied by poly(methyl methacrylate) (PMMA), which was subsequently evaporated to create empty spaces, i.e., the macropores. The size of the macropores can be controlled by adjusting the PMMA size, with a larger PMMA proportion resulting in larger macropores. Furthermore, the porosity of the membrane could be regulated by varying the PMMA:TWC ratio, whereby a higher PMMA proportion yielded greater porosity.

**Figure 7:**
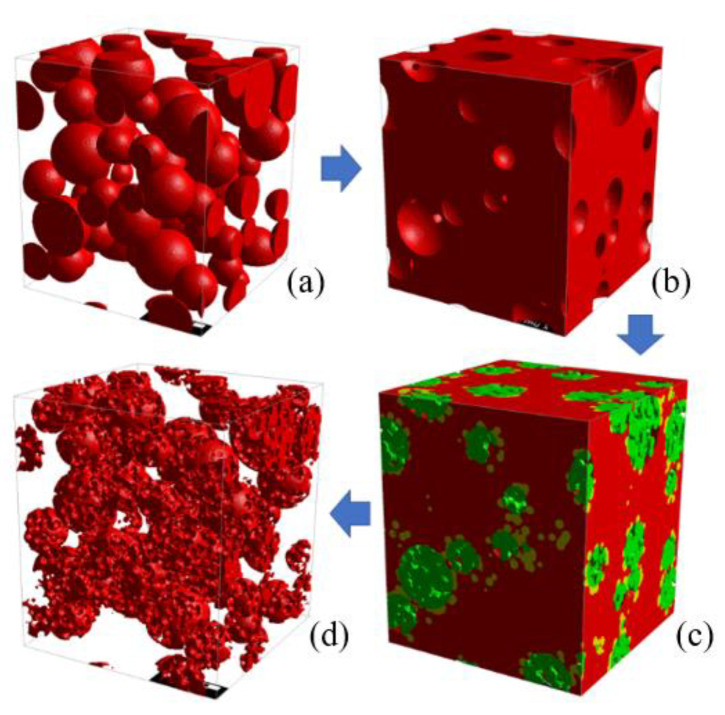
Simulation procedure of porous-particle TWC membrane generating. Create a solid-particle TWC membrane (**a**), revert the domain to create a space for PMMA filtration (**b**), PMMA infiltration (**c**), and revert the domain once again to achieve the macroporous structure of a TWC membrane (**d**).

The porous-particle three-way catalyst (TWC) membrane was optimized in the simulation. The simulation involved adjusting the PMMA size and PMMA:TWC ratio to create a membrane with the desired properties. First, the solid-particle TWC membrane with a diameter of 1 µm to 1.5 µm was generated using the filtering simulation explained in Section 2.2 and Section 2.3. The voxel size was reduced to 12.5 nm to account for the complex shape of the macroporous structure. The original solid-particle membrane had a porosity of approximately 68%. Next, the original domain was reverted to create a space to fill the PMMA particles inside the solid TWC particle, i.e., the pores in the original domain became solid and vice versa. Then, several sizes of PMMA particles ranging from 60 nm to 350 nm were filled into the empty reverted domain. PMMA size is key to defining the size of macropores in TWC particles. The ratio of PMMA:TWC was controlled to adjust the porosity of the domain. Finally, the domain was reverted back, and PMMA particles became macropores encompassing TWC particles. The entire process is illustrated in Figure 7.

To assess the gas transport properties of the porous-particle TWC membranes, airflow was introduced through the porous-particle membrane at a superficial velocity of 3.4 cm/s. A velocity profile was visualized in the cross-sectional horizontal plane perpendicular to the flow direction. The flow characteristics inside the macropores were then analyzed to evaluate gas transport through the interconnected pore network inside the particles. Higher flow rates indicate greater molecular diffusion and higher catalytic activities [18].

## 3. Results and Discussion

### 3.1. Soot Trapping on Membrane

**Figure 8:**
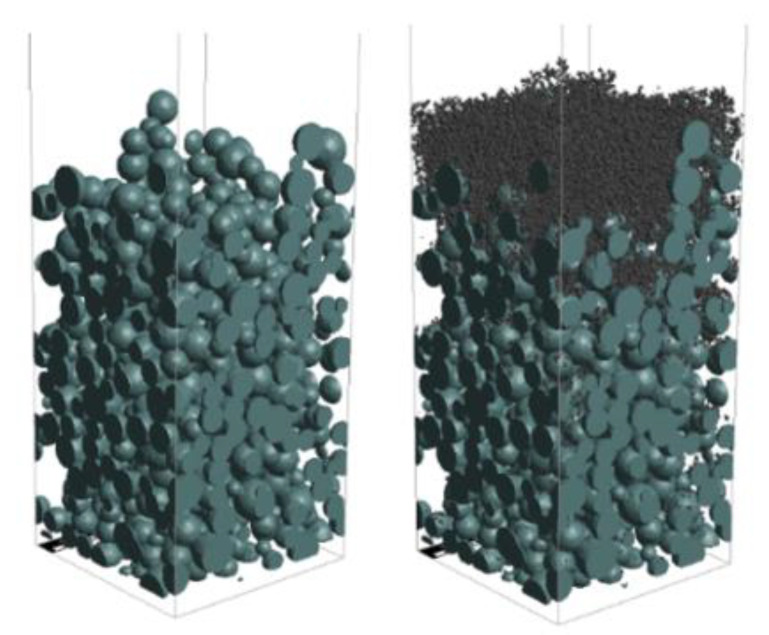
Artificial membrane layer thickness of 30 µm before and after soot deposition.

**Figure 9:**
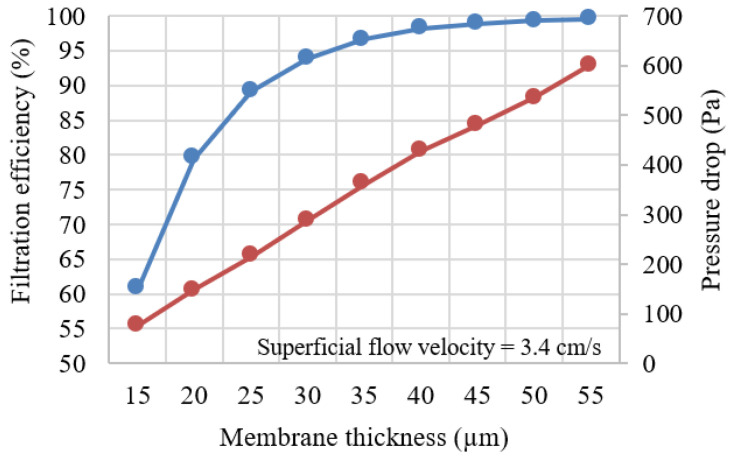
Initial soot filtration efficiency (blue) and initial pressure drop (red) at each membrane thickness.

First, a TWC membrane was fabricated following the procedure outlined in Section 2.2 and Section 2.3. The voxel size was changed to 100 nm to make it suitable for the soot-trapping simulation. The membrane structure consisted of spherical particles with a mean diameter of 1.6 µm and a porosity of 65%. The thickness of the membrane was varied from 15 µm to 55 µm to investigate the effect of membrane thickness on soot trapping. Figure 8 illustrates an artificial membrane layer with a thickness of 30 µm before and after soot deposition. Figure 9 shows the initial soot filtration efficiency (blue line) and the initial pressure drop (red line) for each membrane thickness. At the initial stage, the filtration efficiency increased drastically with the membrane thickness. Later, efficiency reached approximately 90% at a 25 µm membrane thickness. However, after reaching a 30 µm threshold, soot filtration performance only marginally improved until it reached 100% at 55 µm thickness. However, the pressure drop increased linearly with the membrane thickness. 

Thicker membranes exhibited better filtration on the basis of filtration efficiency and pressure drop. However, thicker membranes also resulted in increased pressure drop and material consumption. Consequently, a reference membrane thickness of 30 µm was selected as an optimization point between ensuring filtration efficiency of at least 94% and preventing excessive pressure drop. 

### 3.2. Optimization of Granular Substrate for Membrane Fabrication

**Figure 10:**
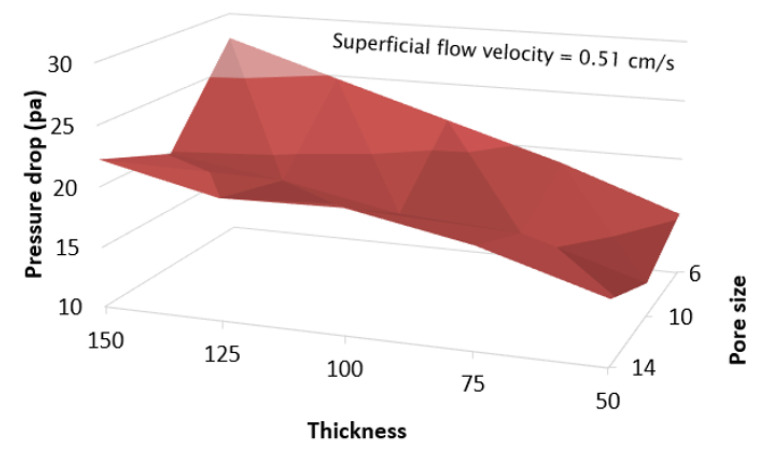
Pressure drop of each support after fabricating a membrane.

Membrane fabrication simulations were performed on 15 support materials. Two parameters were the pore size and the thickness of the support. Three pore sizes were examined, 6, 10, and 14 µm. Each pore size was used for five membrane thicknesses, 50, 75, 100, 125, and 150 µm. As in Section 3.1, the membrane particles were fabricated on a selected support until the coverage reached 99%. After the fabrications were complete, the pressure drops in each support were compared, as shown in Figure 10. These results indicate that as thickness decreased, pressure drop decreased, regardless of pore size. However, there was a local minimum pressure drop at some point between 6 µm and 14 µm pore sizes. This local minimum pressure drop was found at every thickness. Therefore, high-resolution simulations were conducted to investigate each support’s lowest pressure drop and lowest membrane material consumption. 

**Figure 11:**
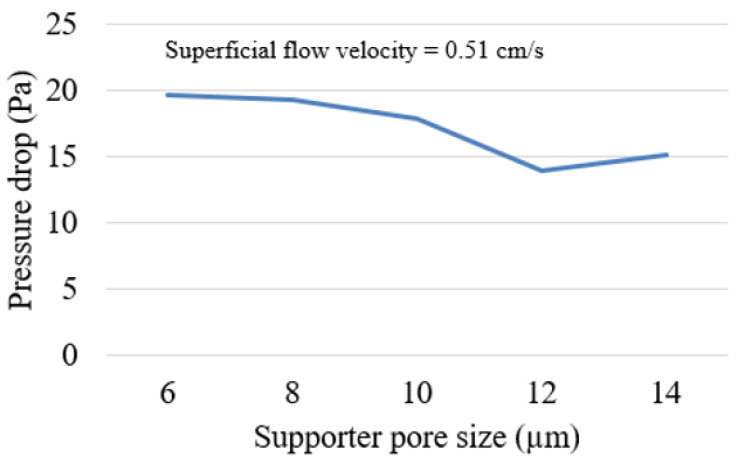
Pressure drop results for a 30 µm membrane fabricated on a 75 µm support with pore sizes from 6–14 µm.

In the simulation, we examined the effect of varying the pore size of the support from 6 µm to 14 µm, which had a fixed thickness of 75 µm. The stopping criteria of the membrane fabrication were changed from the coverage percentage to the total thickness of the membrane at 30 µm, based on previous soot trapping performance in Section 3.1. The pressure drop results for a 30 µm membrane fabricated on each support are depicted in Figure 11. The results demonstrated that the support with the smallest pore size had the highest pressure drop. As the pore size increased, the pressure drop decreased, with a local minimum pressure drop occurring at a pore size of 12 µm. However, at a pore size of 14 µm, the pressure drop began to increase again. With pore sizes larger than 16 µm, the 30 µm membrane could not uniformly cover the support due to its rough surface, resulting in the formation of islands. This might lead to a weak connection within a membrane structure that cannot be compared to other supports using the 30 µm membrane criteria. Therefore, the supports with a pore size larger than 16 µm will require a membrane thicker than 30 µm, which yields an even higher pressure drop and more material consumption. Additionally, the material consumption for the 30 µm membrane fabricated on each support ranged from 35 to 36 g/m^2^, with no significant differences among the cases. Consequently, a pore size of 12 µm appears to be the optimal choice for supporting a 30 µm-thick membrane.

**Figure 12:**
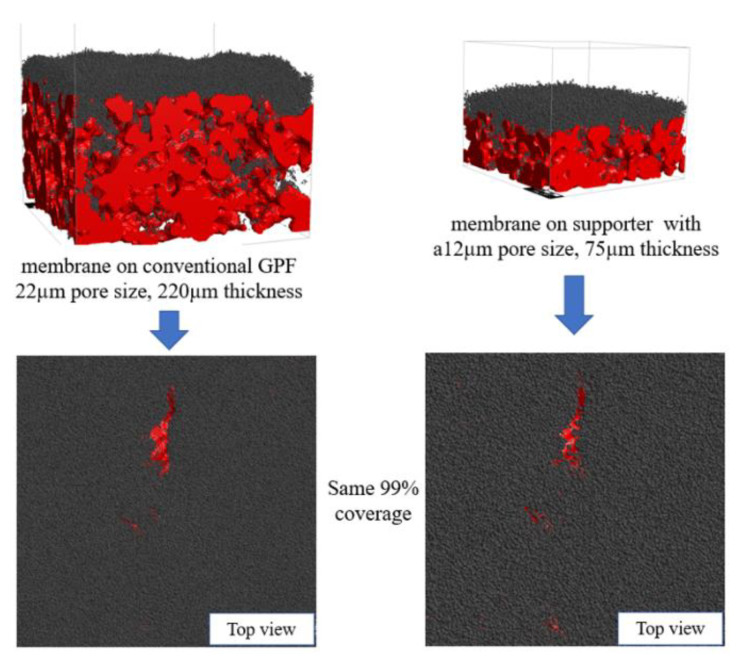
Membrane layer fabricated on a conventional GPF (**left**), and the modified support with a 12 µm pore size and 75 µm thickness (**right**). Both have the same coverage, 99%.

**Figure 13:**
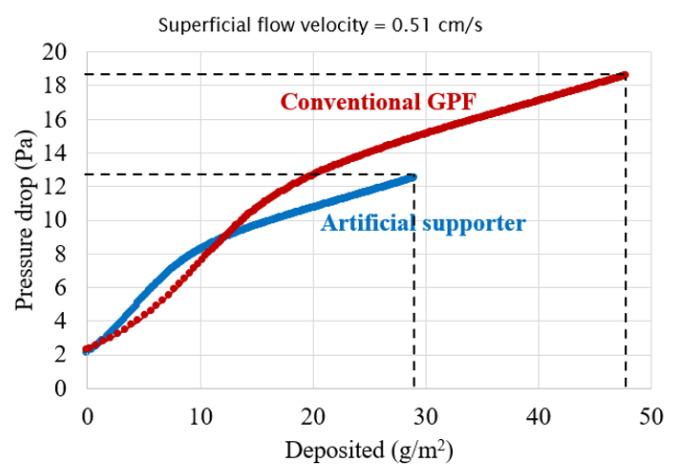
Pressure drop during membrane fabrication on the conventional GPF (red) and the optimized support (blue).

The performance of an optimized support with a 12 µm pore size and 75 µm thickness was evaluated against a conventional GPF by assessing pressure drop and material consumption during membrane fabrication. A significant difference in the pore size of the two supports led to a disparity in the membrane surface morphology. An optimized support with its smaller pore size produced a flat surface membrane that covered the whole substrate much faster. In contrast, conventional GPF produced a rippled membrane that could not cover the substrate at the same membrane volume. As such, the percent membrane coverage was used to evaluate the completion of membrane fabrication, rather than the membrane thickness. During the sintering process, the fully covered membrane yields a strong connection, which plays an important role in the overall durability of the membrane structure [19]. Thus, the criteria for halting membrane fabrication was set at 99% coverage, as shown in Figure 12. The pressure drop during membrane fabrication for each support is illustrated in Figure 13. While the initial pressure drop was comparable for both supports, the optimized support with a narrower pore size led to an earlier completion of pore filtration and a quicker transition to the cake layer deposition phase, as indicated by the linear increase in pressure drop. As a result, the optimized support demonstrated a significant improvement in performance, with a 33% reduction in pressure drop and a 39% decrease in material consumption compared to a conventional GPF.

### 3.3. Optimization of the Porous-Particle Membrane

**Figure 14:**
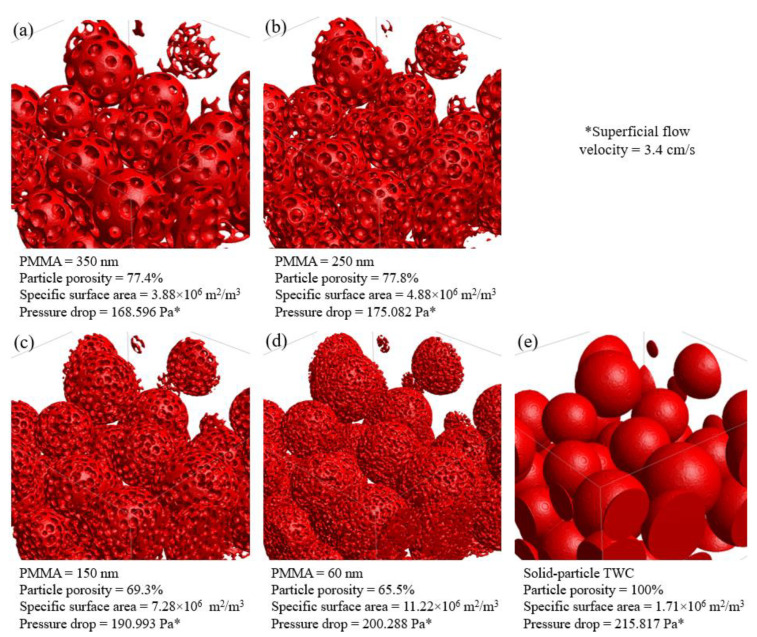
Porous-particle TWC membranes with PMMA sizes of 350 nm (**a**), 250 nm (**b**), 150 nm (**c**), 60 nm (**d**), and A solid-particle TWC membrane (**e**).

**Figure 15:**
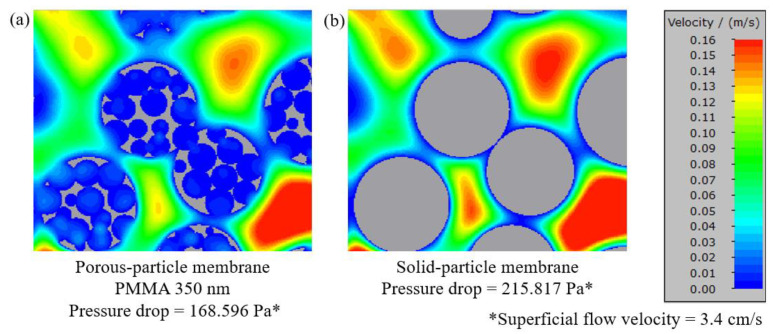
Velocity profile visualization in a cross-sectional horizontal plane of a porous-particle membrane with 350 nm PMMA (**a**) and solid-particle membrane (**b**).

Figure 14a–d present porous-particle TWC membranes with PMMA sizes of 350, 250, 150, and 60 nm, respectively. Figure 14e presents solid-particle TWC membranes with their porosity, specific surface area, and pressure drop specified under each image. After feeding an air flow with a superficial velocity of 3.4 cm/s, all porous-particle membranes exhibited lower pressure drops compared to a solid-particle membrane. This is because the working gas is partially distributed within the interconnected pores inside the particles, leading to a reduced main flow velocity between TWC particles, as illustrated in Figure 15. Consequently, the porous-particle membrane with a 350 nm PMMA pore size produced a significant pressure drop reduction of 21.9% compared to the solid-particle TWC membrane. Additionally, among the various PMMA sizes ranging from 60 nm to 350 nm, the 350 nm PMMA demonstrated the lowest pressure drop. This can be attributed to the larger macropores inside the 350 nm PMMA particles, facilitating better flow distribution and minimizing flow resistance between TWC particles. 

The larger macropores inside the particles also present an advantage in a more significant flow and greatly enhanced catalytic activity [20,21]. However, a larger PMMA had a drawback due to smaller surface area compared to a smaller PMMA. Therefore, airflow simulations through the membranes have been conducted to examine the catalytic activity enhanced by each type of porous structure. One representative particle from each porous-particle TWC membrane candidate was selected to observe the flow through an interconnected pore network inside their macropores. The representative particles were identical in size and location for every domain since they originated from an identical solid membrane. Thus, all domains can be compared since they have the same particle arrangement and morphology.

**Figure 16:**
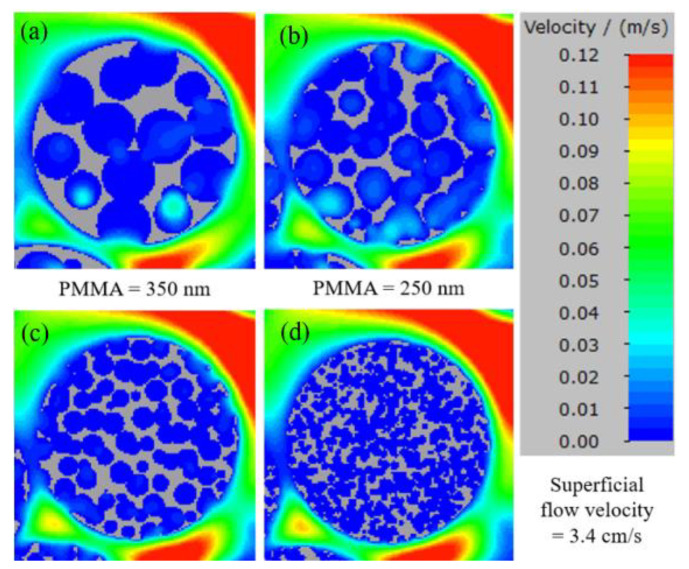
Velocity profile visualization in a cross-sectional horizontal plane at the center of a 1.22 µm particle with PMMA sizes of 350 nm (**a**), 250 nm (**b**), 150 nm (**c**), and 60 nm (**d**).

**Figure 17:**
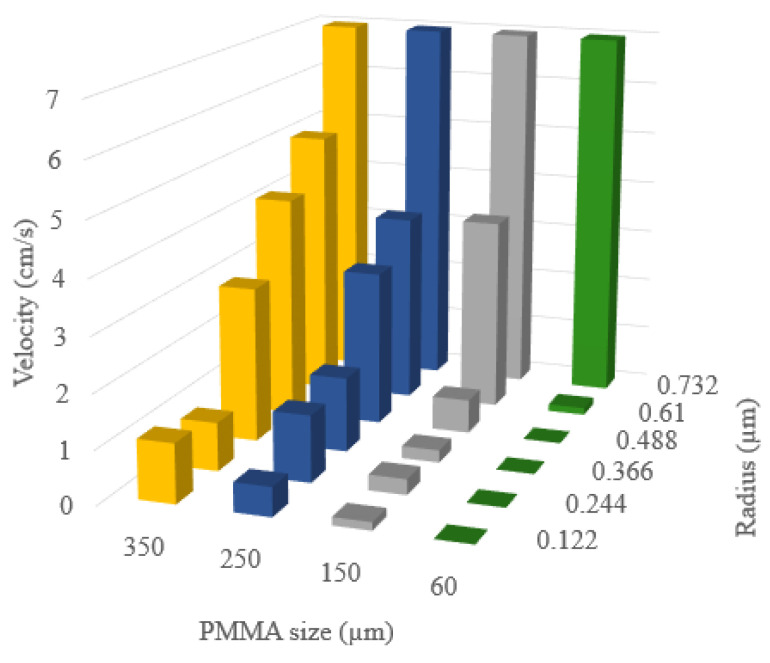
Maximum velocities at each radius layer comparing a porous-particle membrane with PMMA sizes ranging from 60 nm to 350 nm.

To compare the flow characteristics of different PMMA sizes, a velocity profile was visualized in a cross-sectional horizontal plane through the center of a 1.22 µm particle in Figure 16a–d. The particle is divided into six layers from the inner to the outer radius for a clear comparison. The maximum velocities at each radius are depicted in Figure 17. The 0.732 nm radius layer represents velocity close to the particle surface. Here, maximum velocities were the same across all sizes of PMMA. At a 0.61 µm radius, PMMA sizes ranging from 150 nm to 350 nm showed some flow inside the particle. The 350 nm PMMA demonstrated an outstanding flow velocity among all cases. Its maximum internal flow velocity was only 30% less than the surrounding flow velocity outside the particle, whereas the 250 nm and 150 nm-sized PMMA had a maximum flow that was 50% lower. However, the internal flow for the 60 nm-sized PMMA particle was negligible at the 0.61 µm radius layer. This is due to its small constricted pores that create a high-pressure drop. Thus, the flow could not enter the macropores even just below the top surface. At a radius layer at 0.488 µm, the 350 nm PMMA had a faster flow compared to the 250 nm PMMA, and the flow inside the 150 nm PMMA particle was negligible. Additionally, the flow inside the particle drastically dropped for the 250 nm PMMA at a radius of 0.366 µm.

## 4. Conclusions

The simulation results revealed that increasing the membrane thickness improved soot filtration efficiency but also led to a higher pressure drop. Almost 100% filtration can be achieved using a 55 µm thick membrane. For optimization between pressure drop and filtration efficiency, a membrane thickness of 30 µm was selected, ensuring a filtration efficiency of at least 94% while avoiding excessive pressure drop. Optimization showed that a thin granular support was the most favorable option for fabricating a membrane particulate filter due to its lower pressure drop in every scenario. Still, the durability of the support must also be taken into consideration. Among all supports, a 12 µm pore size granular support coated with a 30 µm membrane provided the lowest pressure drop. This approach achieved about 33% lower pressure drop and 39% less material consumption compared to a membrane fabricated on a conventional GPF.

Based on velocity profile analysis, the porous-particle membrane with a 350 nm PMMA contributed the most to the flow through an interconnected pore network inside the particles resulting in the greatest reduction in pressure drop, 21.9%, compared to a solid-particle membrane. In contrast, 60 nm PMMA, which had the largest specific surface area, only influenced molecular diffusion at the outer surface but did not contribute to flow into the macropores. This was due to the small pore size of the 60 nm PMMA, which made it challenging to create an interconnected pore network throughout the entire particle. This resulted in almost no flow entering the particle and the highest pressure drop among all PMMA cases. Therefore, using 350 nm PMMA particles is recommended, based on these findings. They effectively promote the greatest flow through an interconnected pore network and enhance catalytic activity.

## Data Availability

Data sharing is not applicable to this article.
